# Support for the Struggling Learner in Urology

**DOI:** 10.1007/s11934-025-01274-4

**Published:** 2025-05-17

**Authors:** Poojha Prabahara Sundar, Juan Luis Garcia, Natalia Iding, Shannon Cannon

**Affiliations:** 1 University of Wisconsin, Madison, WI USA; 2 University of Wisconsin School of Medicine and Public Health, Madison, WI USA; 3 Department of Urology, University of Wisconsin School of Medicine and Public Health, Madison, WI USA

**Keywords:** Urology residency, Remediation, Graduate medical education, Attrition, Clinical learning environment, Residents

## Abstract

**Purpose of Review:**

Supporting struggling urology residents is an important yet challenging task. We describe strategies to assess resident performance, challenges in the clinical learning environment that contribute to resident remediation, and support mechanisms. This review highlights existing resources for positive outcomes in remediation and promotes exploration of innovative strategies to support future urology residents.

**Recent Findings:**

Burnout is associated with poor resident performance. Factors that promote burnout include unsatisfactory work-life balance, education or financial debt, access to mental health care, and identifying as a member of an underrepresented group. Competency-based assessment and tailored educational interventions can effectively address the needs of a struggling learner. Efforts to create standardized language, specialty-specific remediation programs, and robust mentorship infrastructure have been successful in other specialties.

**Summary:**

Study of interventions and outcomes for struggling urology residents is somewhat limited; deeper understanding of prevailing remediations practices and individual needs of struggling residents will be critical to develop more robust support for trainees.

## Introduction

Residency training is undeniably challenging professionally, personally, physically and emotionally. For most, the challenges that residency presents are not insurmountable. However, a small minority of trainees will not complete residency. This is a costly outcome for the trainee, the program they are leaving, the medical profession they would have entered, and ultimately, for the patient. Residency programs are tasked with ensuring trainees who complete a program are competent and ready for independent practice, yet they are also responsible for identifying and addressing any deficiencies that might impede a residents’ trajectory towards competency. The early identification and management of deficiency is a critical yet challenging duty.

In this narrative review, we focus on the current landscape of remediation and attrition in Urology residency training. We discuss strategies to assess resident performance, challenges in the clinical learning environment that may contribute to resident remediation, and both structural and non-structural approaches to support the struggling resident. The goal of this report is to understand existing resources for remediation and to promote exploration of innovative strategies to support future urology residents.

### Struggling Learners in Urology

Urology is a smaller surgical specialty, with an estimated 14,176 urologic surgeons practicing in the United States (US) [1]. As such, Urology training programs also train fewer trainees compared to general surgery and other larger specialties. In 2024, there were 1,810 active urology residents across 150 programs in the US, with 319 graduating residents [2]. When compared to US population and medical school demographics, Urology has decreased racial, ethnic, and gender diversity. Women comprise 11.8% of the urologic workforce, and 33% of urologic trainees [1, 3]. Black and Hispanic/Latino urologists represent 1.8% and 4.7% of the urologic workforce, respectively; among trainees, 5.2% identify as Black, 8.4% as Hispanic, and 4.5% as multiracial [1, 3].

Attrition is a significant concern for urology residency programs, as it is in other specialties. A recent study of 2001–2016 trainee data suggests a 3.7% attrition rate in Urology residencies, lower than prior estimates in general surgery ranging between 12 and 17% [4]. Attrition disproportionately affects women and trainees who are underrepresented in medicine (URiM). While attrition rates among women have declined, attrition among URiM trainees increased by 125% [4]. These changes are attributed to increasing numbers of women in the field and associated opportunities for mentorship and support; similar increases in URiM urologists have not been observed. Further, increases in URiM attrition in urology was accompanied by a decline in the number of URiM matriculants. Increasing underrepresentation can contribute to feelings of isolation, impact team camaraderie and negatively affect clinical performance [4].

In the 2023-24 academic year, 15 residents left their residency prior to graduation; of these, 6 transferred programs, 7 residents withdrew voluntarily and 2 residents were dismissed from their programs [2]. While attrition is an outcome for a small proportion of urology residents, the number of residents who are “struggling” or who are on informal or formal remediation plans is less clear, but these likely represent a much larger proportion of urology trainees.

The definition of a struggling learner is variable and lacks consensus. The American Board of Internal Medicine previously used the term “problem” resident to describe a trainee who shows enough problem to require intervention by someone of authority, whereas another definition describes a “problem” resident as one who fails to meet at least 1 expectation on the Accreditation Council for Graduate Medical Education (ACGME) core competencies [5, 6]. By these definitions, prevalence rates of struggling learners range between 9–28% [7–9]. Han et al. conducted a national survey of Urology program directors (PDs), and found that of 73 responding PDs, 63% had formally remediated at least 1 resident over the last 5 years, with ”Professionalism” and “Interpersonal and Communication Skills” as the most common deficiencies [10]. Top formal remediation plans included faculty coaching/mentorship (80%), utilizing available wellness resources (74%), and dedicating study time or requiring additional coursework (72%). Additionally, 88% of PDs notified the GME office about formal remediation efforts for trainees [10]. A survey of Emergency Medicine PDs attempted to characterize prevailing terminology and practices [11]. Yet, variability in terms used to describe a struggling learner and the process of escalating remediation, even within a single specialty, highlight the significant limitations to broader study of struggling learners in Graduate Medical Education.

### Measuring Resident Performance

Regular performance assessment is often the initial signal that a resident may be struggling or in need of additional support. The ACGME Milestones were implemented in Urology in 2013 and encompass competencies and sub-competencies that represent the essential and specific skills that must be emphasized within Urology residency [12]. There are 6 competencies: patient care and procedural skills (PC), medical knowledge (MK), professionalism (PROF), systems-based practice (SBP), interpersonal and communication skills (ICS), and practice-based learning and improvement (PBLI) [13]. Residents can progress within each sub-competency, with the goal of reaching milestone Level 4 by graduation and Level 5 reserved for exceptional expertise in a specific area [12]. A struggling resident may not meet milestone expectations in one or more areas, and the approach to remediation should be tailored to the areas of deficiency [14].

While ACGME Milestones provide broad assessment of trainee competence, they do not provide granular or clearly defined competency benchmarks for development of specific surgical skills. The use of competency-based surgical assessments has increased since 2013; however, reliable and standardized assessment tools are still lacking [15]. Though research on competency-based surgical assessments in Urology is limited, studies in Plastic Surgery and Emergency Medicine suggest these models enable longitudinal assessments driven by feedback, offering a comprehensive view of surgical skills [16–18]. Trainees often report low confidence in performing surgeries independently, and increased use of surgical skill assessment tools can help to promote confidence and autonomy [19, 20]. For the resident with specific deficiencies in PC, a focused and specific assessment of surgical skills at higher frequency may aid with progression toward milestone attainment.

Evaluations are not only essential to promote milestone progression and surgical autonomy; they also provide early indications of a resident who is struggling. However, consensus is lacking on the optimal frequency and depth of feedback for it to be effective. A 2024 urology report highlighted a disconnect between PDs and residents regarding feedback quality, with residents wanting improvements in frequency and quality of feedback [21]. Urology programs have explored various evaluation methods to address this low confidence and disconnect between PDs and learners. A 2021 study of the Society for Improving Medical Professional Learning (SIMPL) app, a workplace**-**based assessment tool, showed that the app allowed for more in-depth feedback for urology trainees [22]. As competency-based medical education (CBME) is increasingly adopted in residency training programs, workplace-based assessment tools will enable evaluation of practice readiness and determine whether educational interventions have yielded improvement, while also providing critical feedback for struggling residents [23].

With the rise of precision medicine, the concept of precision medical education (PME) has also been proposed as the next paradigm shift in medical education. PME is defined as “a systematic approach that integrates longitudinal data and analytics to drive precise educational interventions that address the individual learner’s needs and goals in a continuous, timely and cyclical fashion” [24, 25]. Modeled after the P4 precision medicine framework, PME aims for proactive evaluation, personalized educational experiences and assessments, learners participating in the creation of interventions for their own education, and predictive education interventions that center a learner and programs’ desired outcomes [24]. Data analytics and the use of artificial intelligence are considered instrumental to this approach [24]. PME may be combined with competency-based medical education; in the setting of variable-length medical education, this approach could aid with early identification of deficiency and more efficient educational content to improve areas of deficiency. A comprehensive institutional strategy is required to effectively implement PME, but elements of this approach may be helpful for struggling residents.

### Challenges in the Clinical Learning Environment

There are many challenges learners face in the clinical learning environment that may underlie poor resident performance. In particular, poor well-being, often associated with burnout, has been consistently linked to poor resident performance and patient outcomes. A 2022 literature review identified associations between well-being measures (burnout and depression) and medical errors, decline in quality patient care, decreased academic achievements, decreased perceived success, and lower trainee examination scores [26].

With known negative impacts of poor well-being on resident performance and patient outcomes, burnout rates are alarmingly high in urology residents. The 2019 AUA census reported 47% of residents experienced burnout and 53% reported career choice regret [27, 28]. Harris et al. examined urology resident and fellow data from the 2021 AUA census, which included the Maslach Burnout Inventory, a validated questionnaire to assess professional burnout. Among respondents, 48% of residents and 33% of fellows met criteria for burnout, and 74% of residents reported medium to high levels of depersonalization [28]. Additionally, rates of burnout among second-year residents are exceedingly high (70%) [27, 28]. Concerningly, burnout has not decreased over time, with similar rates of trainee burnout observed on the 2021 Census and disproportionate increases in burnout among women in urology by 14% [28, 29].

Many studies have been conducted to investigate the underlying causes of poor well-being and burnout among physicians. These causes include institutional factors and work life balance, financial factors, access to mental health, and personal/cultural factors. Institutional factors often directly impact work-life balance. Among practicing urologists, 17% of the top workplace dissatisfier was not having enough time for personal/family life [29]. Urology trainees report similar stressors: work-life balance dissatisfaction and working over 80 h a week is associated with burnout, while spending time with family is protective against burnout [28, 30, 31]. Basic human care such as access to food, sleeping rooms, and the ability to attend medical and mental health appointments were among the biggest unmet needs for well-being [27, 28]. Other studies of urology residents have similarly linked lack of access to mental health care to burnout [30, 31]. Debt is also an identified risk factor for burnout, with one study reporting that 24% of urologists felt that their educational debt contributed to feelings of burnout [32]. While no studies have examined this association in urology residents, studies of residents in other specialties have also identified associations between debt and burnout [33, 34].

Burnout is reported at disproportionately higher rates among trainees who are from underrepresented or marginalized communities. Practicing urologists who are women report higher rates of work-life conflicts, dissatisfaction with work-life balance, higher rates of burnout, and higher likelihood to have educational debt, among other related factors [35]. Another study similarly found debt was higher for Black and female practicing urologists compared to white, Asian, and male physicians [32]. The experience of being underrepresented in medicine has a significant impact on burnout and wellbeing. Studies of surgical residents have found that exposure to discrimination and harassment based on qualities such as race, gender, and parental status are linked to higher rates of burnout [36, 37]. Imposter syndrome, defined as a condition where high achieving individuals feel their success is undeserved or fraudulent, is observed at high rates among urology residents, with 40% reporting experiencing imposterism; increases in the experience of imposterism were associated with a higher rate of burnout [38]. Similar findings have been described in general surgery residency, imposterism found to be an independent risk factor for both anxiety and burnout [39]. It is important to consider the complexity of factors that may underlie a struggling resident’s performance, and to especially consider the disproportionate effects of these factors on residents from backgrounds that are underrepresented in urology.

While many urologists acknowledge the need for mental health services, barriers to access to these services remain in place for many trainees. One urological study found that residents unable to access care had an 18% higher risk of burnout, with additional challenges for those working over 80 h per week [40]. A 2024 survey of 467 urologists revealed that while many experienced anxiety (85%), guilt (81%), and depression (71%) following surgical complications, most (94%) did not receive counseling [41]. Similarly, a 2022 study of urological PDs revealed that while 75% supported mental health screening for learners, 72.2% expressed concerns about its implementation. Concerns included delays and additional distress from screening results and fears that such outcomes could negatively impact patient care [42]. Studies have emphasized the need for a better understanding of mental health wellness. Active awareness strategies have been highlighted, such as regular burnout assessments, wellness programs, social events, and fostering open discussions with senior surgeons to address mental health challenges [43, 44]. A 2021 AUA burnout survey also found that trainees reported improved mental health through self-care practices like better nutrition, sleep, and healthcare access [27].

Promoting work-life balance is crucial for improving mental health among urology learners. A 2022 survey found that urologists with children under 18 were 35% less likely to report satisfaction with their work-life balance, and satisfaction decreased by 16% for every additional five hours worked per week [45]. Despite the connection between work-life balance and mental health, limited research has explored how family support contributes to achieving this balance in urology. One general surgery study showed a positive correlation between family involvement and social well-being but also found that residents still experienced high burnout despite strong support [46].

### Institutional Support for the Struggling Learner

The task of identifying and supporting a struggling resident is under the purview of the local residency program, as the program itself is ultimately responsible for ensuring a resident has adequately met criteria for graduation. However, at the national level, ACGME provides high-level guidance and specifies resources that residency programs must make available for residents who are at risk or struggling. The ACGME Common Program Requirements (CPR) state that an individual remediation plan, developed by the program director or faculty mentor and resident, can take many forms but must be documented. Further, in more significant situations where the time course of resident progression is altered, the program director must follow institutional policies and procedures to ensure due process (CPR, V.A.1 [47]). Mental Health and Wellness support are similarly broadly mandated: “residents must be given the opportunity to attend medical, mental health, and dental care appointments, including those scheduled during their working hours.” (CPR VI.C.1 [47]). The CPRs acknowledge the increased risk among physicians for burnout, depression, substance use disorder and/or suicidal ideation. The CPRs outline that programs must provide education for both residents and faculty members in identification of these conditions, the means for assisting those who suffer from these conditions, and importantly, access to urgent and emergent mental health care (CPR VI.C.1 [47]). These CPR mandates are reiterated in the ACGME Program Requirements in Urology [48].

Between the broad goals outlined in the CPR and specific limitations set out through institutional GME policies, programs directors must navigate the challenge of creating remediation plans that are tailored to the individual learner, sometimes with minimal resources or practical guidance. Standardization of processes and resources has several advantages: it reduces administrative burden of generating novel program-level resources and can allow program directors to focus on specific needs of the individual learner; it ensures institutional policies are followed in remediation practices; and it reduces perception of subjectivity in the remediation process and stigma for those undergoing remediation. While few institutions have implemented standardized remediation across GME programs, centralized programs such as one created at Rutgers University have been positively welcomed by participating faculty and learners alike [49]. Effective centralized programs focus on core competencies, creating toolkits for professional behaviors such as conduct, accountability, and responsiveness to patients, and standardizing internal training to address external factors like burnout [49]. Centralization promotes transparency, allows for a larger pool of faculty mentors outside of a remediating resident’s residency, and positive outcomes from the remediation process [49]. In a survey of Urology PDs, just over half of GME offices were routinely involved in creating and overseeing formal remediation plans [10]. While lack of faculty feedback and early recognition were most cited barriers to remediation, thematic analysis also identified lack of best practices as a barrier [10].

Specialty-specific remediation resources are a potential avenue to promote standardization of practices. The Council of Residency Directors in Emergency Medicine (CORD-EM) surveyed program directors to create standardized terminology for informal stages of remediation and to gather consensus on elements of a standardized letter of concern and remediation contract [11]. Building upon these efforts, the CORD-EM group created a central repository for remediation resources and a remediation consult service to facilitate access to objective, nonbiased, blinded advice from experts in remediation [50]. In a survey of General Surgery PDs, PDs relied primarily on mentoring to address the needs of struggling residents, but nearly 20% had little to no training in this area and 46% reported that resources available to address these needs were not adequate at their institution [51].

Early identification of a struggling learner is critical to potentially prevent more formal remediation actions. There are well documented barriers to early identification, including lack of formal definition of ‘struggling,’ inadequate/inconsistent contact with faculty, fear of legal reprisal, inadequate documentation of concerns, or an area of struggle being overlooked due to excellence in other domains [14, 51]. Mitigation strategies to prompt earlier identification of the struggling resident include the use of group faculty evaluations, more frequent clinical competency committee reviews, increased documentation of verbal feedback, and using simulations and video-based assessment for various milestone deficiencies [6, 14]. While it is important to identify the area(s) of deficiency to create a tailored, individualized strategy for the individual struggling learner, prior recommendations suggest a stepwise approach, focusing on successful remediation in one area at a time [6].

### Non-GME Resources for the Struggling Learner

Mentorship is an important component of a comprehensive surgical education and of career-long professional development. Most strategies for supporting the struggling learner, either during periods of early intervention or during remediation involve increased contact with a mentor [10]. In a survey of 64 Urology PDs, while 75% of residency programs had a mentorship program, only 5% had official training for faculty mentors, and 38% had no formal requirements for meeting frequency [52]. Mentorship is also an important source of burnout mitigation, which can directly impact capacity of a struggling resident to increase performance [52]. A 2019 national survey found that urology residents in structured mentorship programs experienced lower levels of burnout [53]. A 2021 study in general surgery found that mentorship increased feelings of personal accomplishment among senior residents (PGY4 and PGY5) [54]. A urological survey also found that mentorship is a top priority for trainees when determining their future job prospects after completing their fellowship [55]. However, the presence of a formal mentorship program does not equate to the success of a mentorship program; success of mentorship programs in medical training is rarely measured objectively, or measured at all [53]. Further, studies of characteristics of effective mentorship in the setting of intervention for a struggling resident are limited.

Peer support is an emerging area of focus, and peer support relationships can provide a source of additional support for a struggling resident. University of California, San Francisco (UCSF) piloted formal training in peer support skills for 10 residents and offered condensed curriculum to additional 21 residents, with all participants reporting they were extremely likely to incorporate these skills into their professional life [56]. In a qualitative study of internal medicine interns, Moore et al. sought to understand the contributors to emotional distress and the impact of peer support from senior residents [57]. The authors proposed a model of fluctuating emotional distress during residency and they identified times when distress escalates beyond a coping threshold and becomes disruptive to performance; it was at these times of escalated distress when peer support became critical [57]. Within Urology, the UReTER Mentorship Program (**U**nder**r**epresented **T**rainees **E**ntering **R**esidency) has created opportunity for formal mentorship between current urology trainees and aspiring medical students, focused on students from underrepresented backgrounds [58]. Mentorship relationships developed within pipeline-based programs may potentially translate to peer-support networks as students successfully transition into resident positions and build community amongst trainees for whom cultural and social isolation may increase risk of attrition.

Creating and promoting an inclusive clinical learning environment for urology learners, particularly for those from URiM backgrounds, is paramount. Strategies described in the literature include microaggression training and affinity groups. Despite sparse literature on microaggression training in urology, study of other specialties has highlighted the positive outcomes of microaggression training, with residents recommending trainings to colleagues, citing its relevance and increased confidence in applying training skills in practice [59]. Among anesthesiology and surgery residents who completed a microaggression training workshop at UCSF, 75% strongly agreed the material covered was important to their training, and self-perceived competence in responding to microaggressions increased as a result of participating in the workshop [60]. High-realism simulation for microaggression training offers an important bridge to real-world use of skills and can increase a trainee’s confidence to interrupt witnessed microaggressions [61]. Affinity communities in Urology such as Urology Unbound, Society of Women in Urology, and LatinX in Urology have demonstrated positive impact for trainees. These communities support URiM urologists through mentorship, education, and fostering inclusivity [62, 63].

## Conclusions

Attrition rates from Urology residency are low, but many residents struggle or require some form of remediation during training. Lack of standardized terminology and remediation practices may impede full characterization this cohort of resident in Urology. We identified many contributing factors that increase the risk of struggling or requiring remediation, with burnout being a primary contributor that is influenced by unsatisfactory work-life balance, education or financial debt, access to mental health care, and identifying as a member of a marginalized or underrepresented group. Early intervention is critical to reduce risk of attrition, and a tailored approach to remediation based on area of deficiency and contributing factors is recommended. While the ACGME and local GME may provide useful resources for supporting the resident and program as remediation is pursued, some specialties have found success with creating specialty-specific national-level standardization and best practices for remediation. These could serve as a potential model for GME innovation within Urology as well. Non-structural support, particularly mentorship, is a critical component of supporting a struggling resident. We recommend increased focus on mentor training, formalization and financial support for mentorship programming, and study of efficacy of these programs. Finally, support for the whole resident is increasingly acknowledged through ACGME core program requirements, but the implementation of these requirements may be inconsistent. Further study of the real experiences of the clinical learning environment for all residents, including the struggling ones, may help provide areas for targeted efforts at improvement.

## Key References

•• Gurayah AA, Mohamed AI, Rahman F, Bernstein AP, Asafu-Adjei D, Ezeh UC, et al. The Revolving Door of Residency: Predictors of Residency Attrition for Urology Matriculants Between 2001 and 2016. Urology 2023;177:21–8. Association of American Medical Colleges resident survey data focused on demographics and risk factors for attrition in urology residency.
• Han DS, Badalato GM, Murano TE, Anderson CB. Resident Remediation: A National Survey of Urology Program Directors. J Surg Educ 2024;81:465–73. Survey of 73 (49%) of urology residency program directors to characterize remediation prevalence and processes.
• Harris A, Golan R, Kraft K, North A, Modi P, Meeks W, et al. Burnout in Urological Education: An In-Depth Study of Residents and Fellows in the 2021 AUA Census. Journal of Urology 2024;212:205–12. Study of trainee data from the 2021 American Urological Association Census, focused on burnout rates and key factors associated with burnout.
• Murano T, Kunac A, Kothari N, Hillen M. Changing the Landscape of Remediation: The Creation and Implementation of an Institution-Wide Graduate Medical Education Performance Enhancement Program. Cureus 2023. Description of creation of institutional-wide remediation program.

## Data Availability

No datasets were generated or analysed during the current study.
